# Angiotensin II receptor blockade is associated with preserved muscle strength in chronic hemodialysis patients

**DOI:** 10.1186/s12882-019-1223-3

**Published:** 2019-02-14

**Authors:** Yu-Li Lin, Shu-Yuan Chen, Yu-Hsien Lai, Chih-Hsien Wang, Chiu-Huang Kuo, Hung-Hsiang Liou, Bang-Gee Hsu

**Affiliations:** 1 Division of Nephrology, Buddhist Tzu Chi General Hospital, Hualien, Taiwan; 2 Department of Public Health, Tzu Chi University, Hualien, Taiwan; 3 Division of Nephrology, Department of Internal Medicine, Hsin-Jen Hospital, New Taipei City, Taiwan; 4 School of Medicine, Tzu Chi University, Hualien, Taiwan

**Keywords:** Muscle strength, Angiotensin II receptor blockers, Hemodialysis

## Abstract

**Background:**

Sarcopenia, defined as low muscle mass and strength, is highly prevalent in patients undergoing chronic hemodialysis (HD). However, muscle function and muscle mass do not share the same clinical relevance. In fact, muscle strength was more closely associated with the risk of mortality in chronic HD patients than was muscle mass. Therefore, to identify the risk factors of muscle weakness is vital. Angiotensin II overexpression had been recognized to impair skeletal muscle strength. Accordingly, angiotensin II receptor blockers (ARBs) potentially possess a muscle protective effect. This cross-sectional study aimed to identify the factors associated with low muscle strength and to explore the relationship between ARB use and muscle strength in chronic HD patients.

**Methods:**

A total of 120 chronic HD patients, aged 63.3 ± 13.2 years, were included in this study. Basic characteristics, handgrip strength (HGS), body composition, and nutritional status were assessed, and blood samples for biochemical tests were obtained. We divided these participants into normal- and low HGS groups according to the consensus of the European Working Group on Sarcopenia in Older People (EWGSOP).

**Results:**

We observed that 78 (65.0%) patients had low HGS. In our cohort, we found that height (*r* = 0.653; *P* <  0.001), weight (*r* = 0.496; *P* <  0.001), body mass index (*r* = 0.215; *P* = 0.020), skeletal muscle index (*r* = 0.562; *P* <  0.001), albumin (*r* = 0.197; *P* = 0.032), and serum creatinine (*r* = 0.544; *P* <  0.001) were positively and age (*r* = − 0.506; *P* <  0.001), subjective global assessment (SGA) score (*r* = − 0.392; *P* <  0.001), fractional clearance index for urea (Kt/V) (*r* = − 0.404; P <  0.001) and urea reduction ratio (URR) (*r* = − 0.459; *P* <  0.001) were negatively correlated with HGS. According to our analysis, age (Odds ratio, OR = 1.11, 95% confidence interval, 95% CI = 1.05–1.17, *P* <  0.001), HD duration (OR = 1.01, 95% CI = 1.00–1.02, *P* = 0.010), diabetes (OR = 13.33, 95% CI = 3.45–51.53, *P* <  0.001), Kt/V (OR = 1.61, 95% CI = 1.06–2.46, *P* = 0.027), and SGA score (OR = 1.19, 95% CI = 1.03–1.38, *P* = 0.017) were regarded as independent predictors of low HGS. In contrast, ARB use (OR = 0.25, 95% CI = 0.07–0.93, *P* = 0.039) was independently associated with preserved HGS in chronic HD patients, after adjustment for multiple confounding factors.

**Conclusions:**

Our study is the first report in chronic HD patients to indicate a potentially protective effect of ARB on muscle strength. However, further longitudinal follow-up and intervention studies are needed to confirm this finding.

## Background

Sarcopenia, a syndrome characterized by progressive and generalized loss of skeletal muscle mass and strength defined by the consensus of the European Working Group on Sarcopenia in Older People (EWGSOP), leads to impaired activities of daily living, poor quality of life, and enhanced risk of adverse outcomes, such as falls, bone fractures, disabilities, and death [1–4]. In patients with end-stage renal disease (ESRD), sarcopenia has a high prevalence ranging from 14 to 63% [5–7]. Interestingly, muscle strength and muscle mass, two entities of sarcopenia, do not share the same clinical relevance [8]. In fact, muscle mass is not the only determinant of muscle strength. During the aging process, the decline of muscle strength is much more rapid than the loss of muscle mass [9]. In contrast to reports on the general population, a recent large-scale study delineated that neither low muscle mass nor sarcopenia was significantly associated with mortality in chronic hemodialysis (HD) patients [10], whereas several studies demonstrated a significant association between low muscle strength and high mortality in dialysis patients [11–13]. These findings indicate that muscle strength and muscle mass revealed different impacts on mortality in patients with chronic HD. Therefore, to identify the risk factors of muscle weakness is vital.

Overexpression of angiotensin II has been hypothesized to exert negative impacts, via inhibition of IGF-1/insulin/PI3K/Alt intracellular signaling, downregulation of phospho-Akt, and activation of caspase-3 in skeletal muscle, on skeletal muscle homeostasis [14–16]. Infusion of angiotensin II in rats was observed to induce muscle proteolysis [17], a finding which suggested that angiotensin II receptor blocker (ARB) use may have the potential to prevent muscle strength loss. In fact, additional animal studies confirmed the protective effects of ARB on muscle function and mass loss [18–22]. However, the impact of ARB use on skeletal muscle in patients with chronic kidney disease has never been reported and remains to be clarified.

The purpose of our study aimed to identify the factors that contribute to low muscle strength and to explore the relationship between ARB use and muscle strength in chronic HD patients.

## Methods

### Patients

This is a single-center, cross-sectional study of prevalent HD patients conducted between January and December of 2015. Patients who were older than 20 years and had undergone HD for at least 3 months at a medical center in Hualien were recruited, and patients with malignancy, stroke, acute infection, and amputated limbs, as well as those who were bed-ridden, were excluded from the study. We collected a drug use history by a review of medical records, which included ARB, calcium channel blockers, β-blockers, statins, or fibrates. Diabetes mellitus (DM) was defined on the basis of history or anti-diabetic drugs use, while hypertension was defined on the basis of history or receiving anti-hypertensive agents. Blood pressure was measured before the HD session, using standard mercury sphygmomanometers. All participants signed an informed consent form approved by the Institutional Review Board of Tzu Chi Hospital.

### Low muscle strength definition

Muscle strength was assessed by measuring handgrip strength (HGS), an established method to assess skeletal muscle function in patients with chronic kidney disease [23]. We measured HGS on the arm without vascular access in our patients, using a Jamar Plus Digital Hand Dynamometer (SI Instruments Pty Ltd., Hilton, Australia) with a precision of 1 kg (kg). Patients were instructed to hold the dynamometer in the chosen hand and to squeeze it as hard as they could, with the arm at right angles and the elbow by the side of the body in the upright position. Each measurement was repeated three times, with a rest period of 1 min between measurements, and an average value was recorded. All the measurements of HGS were carried out by the same trained operator before each HD session. According to the EWGSOP criteria, low handgrip strength was defined as HGS less than 30 kg for men and 20 kg for women in the ESRD population [4, 5, 7, 8, 24].

### Body composition measurements

Height and post-HD body weight were measured to calculate body mass index (BMI) according to the equation post-HD body weight (kg) divided by height (square meters). Skeletal muscle mass was assessed via leg-to-leg bioimpedance analysis by using a portable whole-body bioelectrical impedance device (Tanita BC 706 dB, Tanita Corporation, Tokyo, Japan) with the patient in the standing position before each HD session. Skeletal muscle index (SMI) was calculated as skeletal muscle mass/height^2^ (kg/m^2^).

### Biochemical investigations

Blood samples were obtained from each subject, and hemoglobin was measured (Sysmex SP-1000i, Sysmex American, Mundelein, IL, USA). After centrifugation, serum levels of blood urea nitrogen, creatinine, glucose, phosphorus, and C-reactive protein were measured by standard laboratory methods (Siemens Advia 1800, Siemens Healthcare GmbH, Henkestr, Germany). The fractional clearance index for urea (Kt/V) and urea reduction ratio (URR) was calculated using a formal, single-compartment dialysis urea kinetic model. Serum intact parathyroid hormone levels (iPTH) were determined with enzyme-linked immunosorbent assays (ELISA; Diagnostic Systems Laboratories, Webster, Texas, USA).

### Assessment of nutritional status

Nutritional status was assessed by modified quantitative subjective global assessment (SGA), which included 7 variables: weight loss in the past 6 months, change in dietary intake, presence of gastrointestinal symptoms, change in functional capacity, comorbidities, subcutaneous loss of fat, and muscle wasting. Loss of subcutaneous fat was assessed over the triceps, biceps, and fat pads below the eyes, and muscle wasting was assessed on examination of the temples, clavicle and shoulder. Each component was assigned a score from 1 (normal) to 5 (very severe). A final score of 7–35 was assigned to each patient. A higher SGA score represents a more severe degree of malnutrition [25].

### Statistical analysis

Continuous variables were presented as mean ± standard deviation or as median and interquartile range, as appropriate. The Kolmogorov-Smirnov test was applied to test normality. The Student’s independent *t-*test or the Mann-Whitney U test were used for comparing continuous variables, according to the data distribution. Categorical variables were shown as absolute numbers with percentages and analyzed by chi-square test or Fisher’s exact test. Correlations between HGS and continuous variables were analyzed by Pearson’s or Spearman’s rank correlation. Univariate and multivariate logistic regression were used to determine the factors associated with low HGS in chronic HD patients. Statistical analysis was performed using SPSS software (version 19.0; SPSS Inc., Chicago, IL, USA). A *P-*value of less than 0.05 was considered as statistically significant in all analyses.

## Results

### Population characteristics

Of 160 chronic HD patients screened, 120 cases met the inclusion criteria. Among them, 63 were male, and 57 were female. The mean age of the participants was 63.3 years, and 78 (65.0%) patients had low HGS. The clinical and laboratory characteristics of patients with or without low HGS are presented in Table 1. Compared with the normal HGS group, patients with low HGS were older, weighed less, were shorter, and had lower skeletal muscle indexes, lower serum creatinine levels, and higher SGA scores, Kt/V and URR.

**Table 1:** Clinical variables of the 120 hemodialysis patients with or without low handgrip strength

Characteristics	All Patients (*n* = 120)	Normal HGS (*n* = 42)	Low HGS (*n* = 78)	*P*
Age (years)	63.3 ± 13.2	55.0 ± 10.1	67.8 ± 12.5	< 0.001*
HD duration (months)	56.5 (23.7–123.8)	47.4 (21.2–118.7)	59.6 (24.1–125.3)	0.214
SBP (mmHg)	142.6 ± 27.1	142.6 ± 26.1	142.6 ± 27.8	0.990
DBP (mmHg)	77.5 ± 16.6	80.2 ± 16.2	76.0 ± 16.8	0.192
Height (cm)	160.1 ± 8.7	164.0 ± 9.8	158.0 ± 7.3	0.001*
Post-HD body weight (kg)	62.2 ± 14.6	66.8 ± 16.0	59.8 ± 13.3	0.018*
BMI (kg/m^2^)	24.1 ± 4.6	24.6 ± 4.5	23.9 ± 4.7	0.436
SMI (kg/m^2^)	10.5 (8.2–13.8)	12.0 (8.8–15.6)	9.9 (7.5–13.4)	0.012*
Handgrip strength (kg)	22.2 ± 9.7	31.1 ± 7.7	17.2 ± 6.6	< 0.001*
Subjective global assessment	13.5 (10.0–17.0)	11.0 (10.0–14.0)	14.0 (11.0–18.0)	0.002*
Hemoglobin (g/dl)	10.4 (9.7–11.1)	10.5 (9.6–11.2)	10.4 (9.9–11.0)	0.789
Albumin (mg/dL)	4.1 (3.9–4.4)	4.2 (4.0–4.7)	4.1 (3.9–4.3)	0.133
Glucose (mg/dL)	129.0 (106.3–166.8)	123.0 (100.0–143.5)	131.5 (110.0–174.0)	0.058
Blood urea nitrogen (mg/dL)	61.4 ± 14.2	64.0 ± 13.5	60.1 ± 14.4	0.150
Creatinine (mg/dL)	9.6 ± 2.0	10.6 ± 2.2	9.1 ± 1.8	< 0.001*
Phosphorus (mg/dL)	4.7 ± 1.3	4.9 ± 1.3	4.7 ± 1.3	0.492
Intact parathyroid hormone (pg/mL)	206.1 (61.2–449.4)	254.5 (107.2–499.2)	195.0 (55.4–440.7)	0.376
C-reactive protein (mg/dL)	0.3 (0.1–0.9)	0.3 (0.1–0.6)	0.3 (0.1–1.0)	0.389
Kt/V (Gotch)	1.34 ± 0.17	1.29 ± 0.17	1.36 ± 0.17	0.020*
URR	0.73 ± 0.04	0.72 ± 0.04	0.74 ± 0.04	0.017*

Values for continuous variables are given as means ± standard deviation and were tested for significant differences by Student’s *t*-test; variables not normally distributed are shown as medians and interquartile range and were tested by Mann-Whitney U test

*HGS*, handgrip strength; *HD*, hemodialysis; *SBP*, systolic blood pressure; *DBP*, diastolic blood pressure; *SMI*, skeletal muscle index; *Kt/V*, fractional clearance index for urea; *URR*, urea reduction ratio

*P < 0.05 was considered statistically significant

Table 2 depicts a subgroup analysis of HD patients with and without low HGS. Among the 120 participants, 54 (45.0%) patients were aged 65 years or older, 44 (36.7%) patients had DM, and 57 (47.5%) had hypertension. The patients in the low- HGS group were older and more of them had diabetes, while more patients in the normal HGS group took ARB. There were no differences in the distribution of gender; hypertension; or calcium channel blocker, β-blocker, statin, or fibrate use. No angiotensin-converting-enzyme inhibitor or renin inhibitor was consumed in our study population.

**Table 2:** Distribution of hemodialysis patients with or without low handgrip strength in subgroup analysis

Characteristics		Normal HGS group (%)	Low HGS group (%)	*P*
Old age (≥65 years)	No	36 (85.7)	30 (38.5)	< 0.001*
	Yes	6 (14.3)	48 (61.5)	
Gender	Male	26 (61.9)	37 (47.4)	0.130
	Female	16 (38.1)	41 (52.6)	
Diabetes	No	34 (81.0)	42 (53.8)	0.003*
	Yes	8 (19.0)	36 (46.2)	
Hypertension	No	18 (42.9)	45 (57.7)	0.121
	Yes	24 (57.1)	33 (42.3)	
ARB use	No	24 (57.1)	61 (78.2)	0.015*
	Yes	18 (42.9)	17 (21.8)	
CCB use	No	20 (47.6)	51 (65.4)	0.059
	Yes	22 (52.4)	27 (34.6)	
β-blocker use	No	26 (61.9)	58 (74.4)	0.156
	Yes	16 (38.1)	20 (25.6)	
Statin use	No	39 (92.9)	64 (82.1)	0.105
	Yes	3 (7.1)	14 (17.9)	
Fibrate use	No	36 (85.7)	68 (87.2)	0.822
	Yes	6 (14.3)	10 (12.8)	

Data are expressed as number of patients, and analysis was performed using the chi-square test or Fisher’s exact test

ARB, angiotensin II receptor blockers; CCB, calcium channel blockers

### Factors correlated with HGS

The correlation between HGS and clinical variables in these HD patients, analyzed by Pearson’s or Spearman’s rank correlation test, is shown in Table 3. HGS was observed to be positively correlated with height (*r* = 0.653; *P* <  0.001), post-HD body weight (*r* = 0.496; *P* <  0.001), BMI (*r* = 0.215; *P* = 0.020), SMI (*r* = 0.562; P <  0.001), albumin (*r* = 0.197; *P* = 0.032), and serum creatinine (*r* = 0.544; *P* <  0.001), while it was inversely correlated with age (*r* = − 0.506; *P* <  0.001), SGA score (*r* = − 0.392; *P* <  0.001), Kt/V (*r* = − 0.404; *P* <  0.001) and URR (*r* = − 0.459; *P* <  0.001).

**Table 3:** Correlation between HGS and clinical variables among 120 hemodialysis patients

Variables	HGS (kg)	
	r	*P*
Age (years)	−0.506	< 0.001*
HD duration (months) ^a^	− 0.144	0.120
Height (cm)	0.653	< 0.001*
Post-HD body weight (kg)	0.496	< 0.001*
BMI (kg/m^2^)	0.215	0.020*
SMI (kg/m^2^) ^a^	0.562	< 0.001*
SGA ^a^	− 0.392	< 0.001*
Hemoglobin (g/dl) ^a^	0.022	0.811
Albumin (mg/dL) ^a^	0.197	0.032*
Glucose (mg/dL) ^a^	− 0.126	0.173
Creatinine (mg/dL)	0.544	< 0.001*
Phosphorus (mg/dL)	0.059	0.527
Intact parathyroid hormone (pg/mL) ^a^	0.009	0.919
C reactive protein (mg/dL) ^a^	− 0.044	0.637
Kt/V (Gotch)	−0.404	< 0.001*
URR ^a^	− 0.459	< 0.001*

HGS, handgrip strength; HD, hemodialysis; SMI, skeletal muscle index; Kt/V, fractional clearance index for urea;

URR, urea reduction ratio

^a^The data showed a skewed distribution and were analyzed by Spearman’s correlation analysis

*P < 0.05 was considered statistically significant

### Factors associated with low HGS

Table 4 delineates the results of logistic regression analysis regarding the factors associated with low HGS. Univariate logistic regression analysis showed that age, height, post-HD body weight, ARB use, diabetes, SMI, Kt/V, and SGA score were significantly associated with the presence of low HGS. Multivariate logistic regression analysis showed that age (Odds ratio, OR = 1.11, 95% confidence interval, 95% CI = 1.05–1.17, *P* <  0.001), HD duration (OR = 1.01, 95% CI = 1.00–1.02, *P* = 0.010), diabetes (OR = 13.33, 95% CI = 3.45–51.53, *P* <  0.001), Kt/V (OR = 1.61, 95% CI = 1.06–2.46, *P* = 0.027), and SGA score (OR = 1.19, 95% CI = 1.03–1.38, *P* = 0.017) were independently associated with low HGS. Meanwhile, ARB use (OR = 0.25, 95% CI = 0.07–0.93, *P* = 0.039) was found to be independently related to preserved HGS in HD patients, after adjustment for age, gender, height, post-HD body weight, HD duration, diabetes, SMI, Kt/V, and SGA score. Thus, our patients with ARB use showed a 75% decreased odds in handgrip strength weakness, when compared with patients without ARB use.

**Table 4:** Logistic regression analysis of the factors correlated with low handgrip strength in 120 hemodialysis patients

Variable	Univariate		Adjusted	
	OR (95% CI)	*P*	OR (95% CI)	*P*
Age (years)	1.10 (1.05–1.14)	< 0.001*	1.11 (1.05–1.17)	< 0.001*
Female	1.80 (0.84–3.87)	0.132	0.42 (0.07–2.44)	0.336
Height (cm)	0.92 (0.87–0.96)	0.001*	0.94 (0.84–1.06)	0.327
Post-HD body weight (kg)	0.97 (0.94–0.99)	0.015*	1.03 (0.92–1.16)	0.590
HD duration (months)	1.00 (1.00–1.01)	0.234	1.01 (1.00–1.02)	0.010*
ARB use	0.37 (0.17–0.84)	0.017*	0.25 (0.07–0.93)	0.039*
Diabetes	3.64 (1.50–8.87)	0.004*	13.33 (3.45–51.53)	< 0.001*
SMI (kg/m^2^)	0.91 (0.83–0.99)	0.029*	0.98 (0.66–1.45)	0.917
Kt/V (per 0.1 increment)	1.34 (1.04–1.73)	0.023*	1.61 (1.06–2.46)	0.027*
SGA	1.14 (1.04–1.26)	0.008*	1.19 (1.03–1.38)	0.017*

*P < 0.05 is considered statistically significant in univariate and multivariate logistic regression analysis (adopted factors: age, gender, height, post-HD body weight, HD duration, ARB use, diabetes, SMI, Kt/V, and SGA)

HD, hemodialysis; ARB, angiotensin II receptor blockers; SMI, skeletal muscle index, Kt/V, fractional clearance index for urea; SGA, subjective global assessment

## Discussion

In this cross-sectional study, nearly two-thirds (65.0%) of our chronic HD patients were demonstrated to have low HGS, according to the EWGSOP definition. Height, weight, BMI, SMI, albumin, and serum creatinine were found to be positively correlated with HGS, while age, SGA score, and Kt/V were negatively correlated with HGS. After multivariable adjustment, age, HD duration, diabetes, Kt/V, and SGA score remained as independent predictors of low HGS. More interestingly, a significantly higher proportion of chronic HD patients with preserved HGS took ARBs, and this association persisted after adjustment for multiple confounding factors.

Angiotensin II overexpression was known to not only increase the catabolic effects on skeletal muscle by inhibiting the Akt/mammalian target of rapamycin (mTOR) pathway but also to induce skeletal muscle protein degradation through reactive oxygen species accumulation, which in turn activates nuclear factor kappa B (NF-κB) and p38MAPK (p38 mitogen-activated protein kinase) [16]. Losartan had been demonstrated to reduce muscle fibrosis and to improve muscle function after cardiotoxin-induced muscle injury by modulation of TGF-β pathway and also to protect against loss of muscle mass in treated immobilized mice, an effect mediated by increased activation of the Akt/mTOR pathway [22]. Bedair et al. showed that losartan-treated mice exhibited a dose-dependent enhancement of muscle healing and muscle regeneration after laceration injury [20]. Furthermore, treatment with irbesartan after cryoinjury also improves muscle repair and regeneration through suppression of the C1q-Wnt/β-catenin signaling pathway [18]. Despite cumulative evidence provided by animal studies, whether ARB use has a protective effect on skeletal muscle in humans is largely unknown. To the best of our knowledge, this association between ARB use and preserved muscle strength in ESRD patients has not yet been reported. We found that ARB use in our chronic HD patients could reveal a 75% decreased odds in handgrip weakness, when compared with patients who did not use ARB. Although this finding appears to be prominent and promising, an unmeasured confounding bias is still possible. Further studies are encouraged to evaluate whether ARB use protects against muscle strength loss in chronic HD patients.

Besides age and sex, comorbidities and nutritional status were well known to be associated with muscle strength in patients with ESRD [26–31]. In our study, the presence of DM is the major predictor of low handgrip strength, which was consistent with the findings of previous studies. DM is associated with accelerated muscle weakness due to rapid skeletal muscle protein breakdown contributed by insulin resistance and increased levels of inflammatory cytokines. Moreover, diabetic neuropathy also has major negative impacts on HGS [28, 32]. Our data showed that low handgrip strength was associated with poor nutrition status, which was also confirmed by other studies [30, 31]. In addition, both Silva et al. and Wang et al. regarded HGS as an indicator of nutritional status in dialysis patients [29, 33].

Our result showed that both HD duration and Kt/V were negatively associated with muscle strength. However, a correlation between HGS and Kt/V was not found in other studies [30, 34]. Dialysis treatment per se has been shown to stimulate protein degradation and increase nitrogen losses [35, 36]. In addition, several studies consistently showed that hemodialysis leads to an increase in skeletal muscle net protein catabolism [37–39], which may partially explain our finding. Meanwhile, patients with malnutrition and small body size can contribute to an overestimation of Kt/V other than dialysis exposure per se. Therefore, high Kt/V in the study might actually imply the small body size or malnutrition status of our patients. Indeed, extremely high Kt/V was observed to be associated with increased mortality in dialysis patients [40, 41]. Unfortunately, standardized creatinine clearance, which was corrected for body surface area, was not available in our study. Further longitudinal studies are needed to clarify the impact of delivered dialysis dose on muscle strength loss.

There are several limitations to our study. First, our sample size was relatively small. Second, the normal value of HGS in the ESRD population remains to be established. The diagnostic criteria for low muscle strength in the geriatric population were adopted in this study; however, whether this cut-off for HGS could be applied to patients with ESRD has not been validated. Finally, a causal relationship cannot be obtained from this cross-sectional study, and our result should be regarded as hypothesis generating.

## Conclusions

We conclude that age, HD duration, diabetes, Kt/V, and SGA score are independent predictors of low HGS, while ARB use is reported for the first time to be associated with preserved HGS in chronic HD patients. However, longitudinal observational follow-up and intervention studies are needed to clarify the role of ARB use in the maintenance of skeletal muscle strength and mass in dialysis patients.

## Abbreviations

ARB: angiotensin II receptor blocker
BMI: body mass index
DM: diabetes
ESRD: end-stage renal disease
EWGSOP: European Working Group on Sarcopenia in Older People
HD: hemodialysis
HGS: handgrip strength
iPTH: intact parathyroid hormone
Kt/V: fractional clearance index for urea
mTOR: mammalian target of rapamycin
NF-κB: nuclear factor kappa B
p38MAPK: p38 mitogen-activated protein kinase
SGA: subjective global assessment
SMI: skeletal muscle index
